# Efficient and stable production of Modified Vaccinia Ankara virus in two-stage semi-continuous and in continuous stirred tank cultivation systems

**DOI:** 10.1371/journal.pone.0182553

**Published:** 2017-08-24

**Authors:** Felipe Tapia, Ingo Jordan, Yvonne Genzel, Udo Reichl

**Affiliations:** 1 International Max Planck Research School for Advanced Methods in Process and Systems Engineering, Magdeburg, Germany; 2 Max Planck Institute for Dynamics of Complex Technical Systems, Magdeburg, Germany; 3 ProBioGen AG, Berlin, Germany; 4 Bioprocess Engineering, Otto-von-Guericke-University Magdeburg, Magdeburg, Germany; University of Applied Sciences Mittelhessen, GERMANY

## Abstract

One important aim in cell culture-based viral vaccine and vector production is the implementation of continuous processes. Such a development has the potential to reduce costs of vaccine manufacturing as volumetric productivity is increased and the manufacturing footprint is reduced. In this work, continuous production of Modified Vaccinia Ankara (MVA) virus was investigated. First, a semi-continuous two-stage cultivation system consisting of two shaker flasks in series was established as a small-scale approach. Cultures of the avian AGE1.CR.pIX cell line were expanded in the first shaker, and MVA virus was propagated and harvested in the second shaker over a period of 8–15 days. A total of nine small-scale cultivations were performed to investigate the impact of process parameters on virus yields. Harvest volumes of 0.7–1 L with maximum TCID_50_ titers of up to 1.0×10^9^ virions/mL were obtained. Genetic analysis of control experiments using a recombinant MVA virus containing green-fluorescent-protein suggested that the virus was stable over at least 16 d of cultivation. In addition, a decrease or fluctuation of infectious units that may indicate an excessive accumulation of defective interfering particles was not observed. The process was automated in a two-stage continuous system comprising two connected 1 L stirred tank bioreactors. Stable MVA virus titers, and a total production volume of 7.1 L with an average TCID_50_ titer of 9×10^7^ virions/mL was achieved. Because titers were at the lower range of the shake flask cultivations potential for further process optimization at large scale will be discussed. Overall, MVA virus was efficiently produced in continuous and semi-continuous cultivations making two-stage stirred tank bioreactor systems a promising platform for industrial production of MVA-derived recombinant vaccines and viral vectors.

## Introduction

Similar to the manufacturing of antibodies, growth factors or hormones, the production of viral vaccines and vectors for gene therapy in batch cultivation is still the standard technology. Moving from batch to continuous operation can significantly increase productivity, improve the quality of products, and reduce the footprint of equipment and facilities required for production [1]. The upstream phase of continuous processes is faster and more efficient compared to batch processes because smaller bioreactors are required, volumetric yields are higher and plant down-time is reduced [2]. Hence, continuous virus production may also help to increase significantly the manufacturing capacity and to meet the global demand for low cost viral vaccines of an ever-growing world population.

One potential platform for continuous production of viruses is a two-stage stirred tank bioreactor (TSB) system. A TSB system consists of one vessel for continuous propagation of cells connected in series with a second vessel, where the desired product is obtained [3, 4]. The physical separation of the cell growth vessel from the production bioreactor allows stable operation of processes involving lytic viruses. TSB systems have been used for the production of poliovirus and adenovirus [4], for production of recombinant proteins using baculovirus [5], and for continuous production of influenza A/PR/8/34 virus [6]. In the latter, virus production was maintained over a time period of 18 d but process yields were lower than for batch cultivations due to oscillations of virus titers. These oscillations were generated by the accumulation of defective interfering particles (DIPs) that spontaneously arise due to error-prone viral replication of RNA viruses with high mutation rates. DIPs are replication-deficient and require co-infections with wildtype or helper viruses for successful propagation. At very high multiplicity of infection (moi), the replication capacity of the helper viruses is limited by DIPs which is known as “passage effect” or “Von Magnus effect” [5–8].

One virus that has a great potential for expression of recombinant antigens or as a viral vaccine vector is Modified Vaccinia Ankara (MVA) virus, a DNA virus that can accommodate large recombinant inserts and is described to be safe for humans and animals [9]. Recombinant vector vaccines based on MVA against influenza virus [10], Ebola virus [11, 12], HIV [13], tuberculosis [14], chykungunya virus [15], smallpox [16], respiratory syncytial virus [17], malaria [18, 19], bluetongue virus [20], West Nile virus [21], and also veterinary vaccines against the tick-born parasite *Babesia bovis* [22], have been described. One challenge in MVA production is the property that a large fraction of infectious units remain cell-associated. Because cultivation in a single-cell format interferes with the spread of MVA, processes have been developed where the virus is propagated in suspended aggregates of 20–100 cells [23]. Recently, a novel MVA virus isolate, named MVA-CR19, has been generated that can be produced at high yields in non-aggregated avian suspension cells in chemically defined media [24], which makes MVA an interesting candidate for exploring process options towards continuous vaccine manufacturing.

When a fraction of a culture is replaced with fresh media at regular time intervals, the mode of operation is defined as “semi-continuous”. Compared to fully continuous operation in bioreactors, a scale-down of semi-continuous cultures can be easily achieved for studying the impact of process parameters on cell growth and virus propagation. In addition, semi-continuous cultures can be set to medium exchange regimes that can approach continuous cultivations [25]. To name one recent example, semi-continuous cultivations using shake flasks have been used for propagation of the rat hybridoma cell line TFL-P9 [26] to evaluate to what extent semi-continuous cultures approach growth kinetics in continuous cultivations. According to this study, kinetic parameters obtained in batch and semi-continuous cultivations of TFL-P9 correlated well with those of continuous cultures. In addition, semi-continuous modes of operation have been used for two-stage production of baculovirus-based recombinant proteins resulting in high yields [27].

Here, we describe results for the establishment of semi-continuous and continuous processes for production of MVA virus (MVA-CR19, and a recombinant MVA virus that expresses green-fluorescent-protein (GFP), MVA-CR19.GFP) in the avian suspension cell line AGE1.CR.pIX in a chemically defined medium (all from ProBioGen AG, Germany) [28–30]. First, a small-scale two-stage semi-continuous cultivation (SSC) system using shaker flasks was established for cell growth and virus propagation at exchange rates that approximated the dilution rates of continuous TSB systems. The impact of process parameters such as the residence time (RT) and the addition of fresh medium on virus yields were analyzed. Furthermore, by using MVA-CR19.GFP, recombinant protein expression and viral stability at the genetic level was evaluated over a time period of 15 d of cultivation. Finally, cell growth, glucose and lactate concentrations, pH values and virus yields were monitored in a TSB system (two 1 L stirred tank bioreactors), and compared to SSC cultivations. The observed genetic stability and high infectious titers demonstrate the feasibility for highly efficient continuous production of MVA viruses.

## Materials and methods

### Cell line and Modified Vaccinia Ankara virus genotypes

The avian cell line AGE1.CR.pIX (ProBioGen AG, Germany) was grown in a chemically defined medium CD-U3 (powder-based, PAA, Austria; liquid, Biochrom-Merck, Germany) supplemented with 2 mM of L-glutamine (Sigma-Aldrich, Germany), 2 mM L-alanine (Fluka Analytical, Sigma-Aldrich, Germany) and 10 ng/mL Long^®^R^3^IGF-I (SAFC Biosciences, USA). Cells were inoculated at a concentration of 0.8×10^6^ cells/mL and passaged in shaker flasks (125 mL Erlenmeyer culture flasks, 2 μm vent cap with baffles; Corning, USA) at 37°C, 5% CO_2_ in air, and 185 rpm [31]. Previous studies have shown that this cell line has stable growth characteristics and can be used for MVA virus production [29,31]. The MVA virus isolates MVA-CR19 (virus seed: 4.5×10^8^ virions/mL, TCID_50_) and MVA-CR19.GFP (virus seed: 1.0×10^9^ virions/mL, FFU; 1×FFU = 0.7×TCID_50_) with a green-fluorescent-protein insertion cassette were used (both isolates from ProBioGen AG, Germany). Before infection, the virus seed was sonicated in a water bath at room temperature for 1 min. An moi of 0.05 (ratio of the number of infectious virions to the number of cells) was used in all cultivations.

### Batch and semi-continuous cultivations for MVA propagation

AGE1.CR.pIX cells were cultured in 50 mL working volume (wv) shaker flasks (Corning, USA), for batch cultivations. With cell concentrations reaching about 5×10^6^ cells/mL [31], a 1:1 dilution with fresh CD-U3 medium was performed. Then, virus infection was carried out and 4–5 mL samples at 0, 12, 24, 36, 48, 72 and 96 h post infection (p.i.) were taken. Cell concentration, cell viability, metabolite concentrations, pH offline and virus titers were determined.

**Fig 1:**
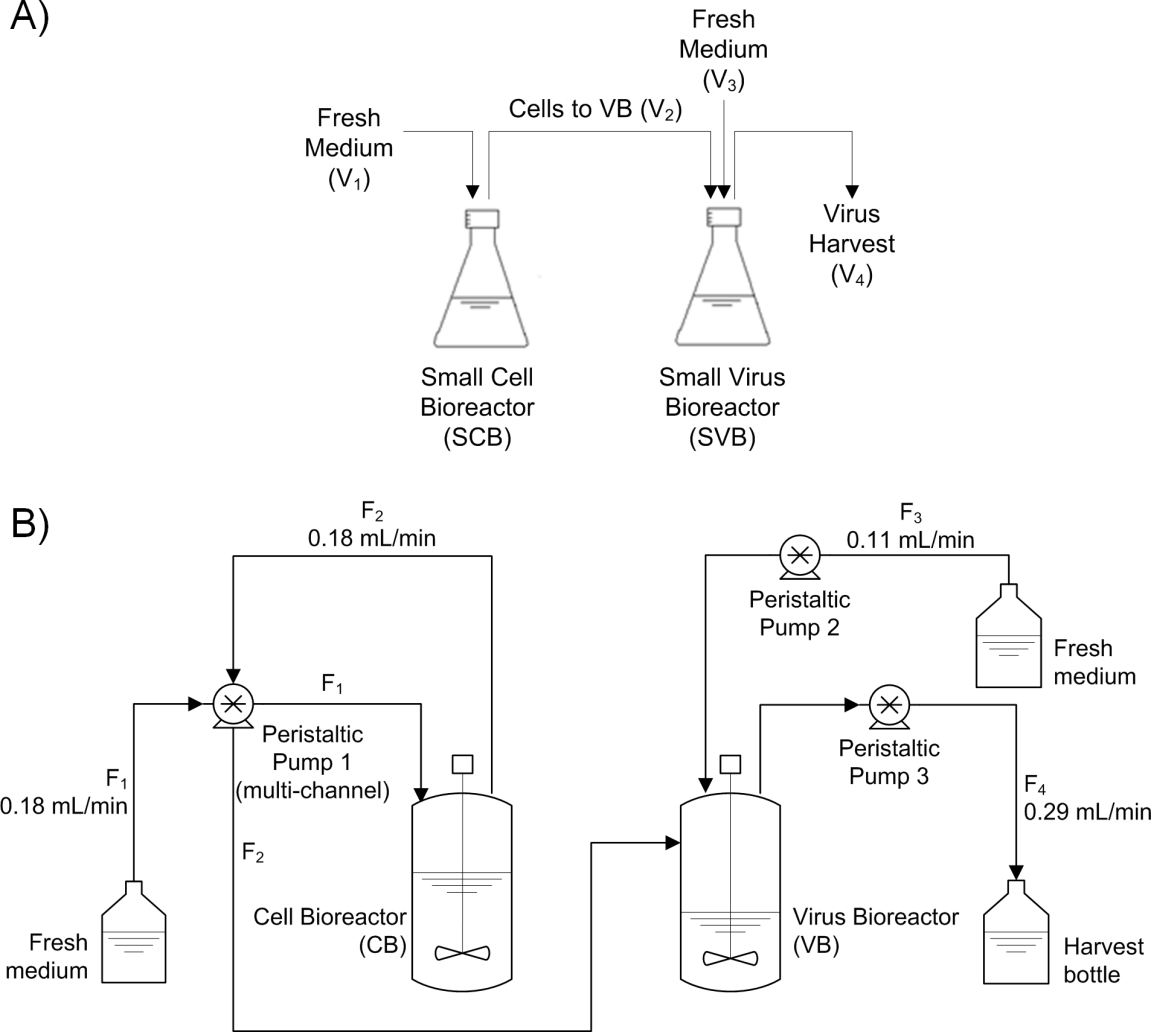
Two-stage cultivation systems used for semi-continuous and continuous MVA virus production. A) Two-stage semi-continuous cultivation (SSC) system for small-scale MVA production using shaker flasks. Cells were produced in semi-continuous mode in the Small Cell Bioreactor (SCB; working volume 120 mL) and transferred to the Small Virus Bioreactor (SVB; 65, 120 or 200 mL working volume), where virus infection and propagation took place. Twice a day, a semi-continuous harvest was taken (V_4_), cells were transferred from SCB to SVB (V_2_), and fresh medium was added to SCB and SVB (V_1_ and V_3_). The volumes of harvest, cell transfer and fresh medium were determined with Eqs 1–4 (see Materials and Methods). Shakers without baffles were used. B) Two-stage continuous stirred tank bioreactor (TSB, 1 L Sartorius Biostat B plus; the working volume of CB and VB was 850 and 440 mL, respectively) system using AGE1.CR.pIX cells with a production flow rate (F_4_) of 0.29 mL/min. Continuous cell production was maintained in the first bioreactor (Cell Bioreactor, CB). Cells were continuously transferred at a flow rate (F_2_) of 0.18 mL/min to a second vessel (Virus Bioreactor, VB; dilution rate 0.0390 h^-1^), where MVA virus infection and propagation took place. Fresh medium was added to CB and VB at a flow rate of 0.18 (F_1_) and 0.11 mL/min (F_3_), respectively.

A SSC system using two shaker flasks (250 mL and 125 mL Erlenmeyer Culture Flasks with a 2 μm vent cap and without baffles; Corning, USA) in series was established as a scale-down model for design and parameter studies regarding TSB optimization. All related abbreviations, volumes and the operation mode of the system are described in Fig 1. The shakers were maintained in an orbital shaking incubator (Infors HT Multitron, Switzerland) at 37°C, 185 rpm and 5% CO_2_ in air. Cells were grown in batch mode for up to 96 h and infection was carried out at an moi of 0.05. Samples of 3–5 mL for measuring cell concentration, viability, pH, metabolite concentrations, and virus concentration in the small cell bioreactor (SCB) and the small virus bioreactor (SVB) were taken twice a day for process monitoring. Small volume losses after sampling were corrected by adding fresh medium. After sampling, medium exchanges and addition of fresh medium were carried out. The volumes exchanged were determined with the following equations:

$$V_{1}={V_{FM\;to\;SCB}}_{n}=(t_{n}-t_{n-1})\times V_{SCB}\times D_{SCB}$$ (1)

$${{V_{2}=V}_{SCB\;to\;SVB}}_{n}=(t_{n}-t_{n-1})\times V_{SCB}\times D_{SCB}$$ (2)

$${{V_{3}=V}_{FM\;to\;SVB}}_{n}=(t_{n}-t_{n-1})\times ((V_{SCB}+V_{SVB})\times D_{SCB}-V_{SCB}{\times D}_{SCB})$$ (3)

$${{V_{4}=V}_{Harvest}}_{n}=(t_{n}-t_{n-1})\times (V_{SCB}+V_{SVB})\times D_{SCB}$$ (4)

Where n is the sample number, V is volume, t_n_ is the time at sampling “n”, t_n-1_ the time at sampling “n-1”, D_SCB_ is the dilution rate of SCB, and FM is fresh medium. The dilution rate of SCB (D_SCB_) was the same in all SSC experiments. The equations were obtained by performing a material balance, where the dilution rate of the TSB system F_4_/(V_CB_ + V_VB_) is equal to the dilution rate of CB (see Fig 1B). Based on previous experiments [31], a maximum specific cell growth rate of 0.02 h^-1^ for AGE1.CR.pIX was used to determine the parameters for all SSC cultivations. Due to the significant amount of samples taken and time required for one SSC run (2 weeks, approximately 36 samples) compared to batch cultivations (7 d, approximately 12 samples) only one cultivation was carried out per SSC experiment (unless the opposite is indicated).

### Evaluation of the influence of process variables on virus yields and virus stability

To evaluate the influence of RT on MVA-CR19 virus yields, the volume of SVB was modified to obtain a RT of 25, 35, and 64 h in the SVB (keeping all other variables constant). Also, the effect of omitting the addition of fresh medium to the VB (F_3_, Fig 1B) was evaluated for one SSC cultivation by reducing V_3_ to zero (Fig 1A). The stability of MVA-CR19 virus was done using the MVA-CR19.GFP recombinant strain under two criteria. The first criterion was that, if the recombinant virus is stable, then the ratio of the total infectious virus population (IVP) to the protein-expressing infectious virus population (PEIVP) should be constant during cultivation time. The PEIVP was measured by determining a GFP-derived TCID_50_ by fluorescence microscopy (described in section 2.4.2). This ratio was determined with the following equation:

$${Ratio}_{TCID50}=\frac{IVP}{PEIVP}$$ (5)

With IVP and PEIVP the mean of a technical triplicate. The second criterion used was a genetic analysis of the GFP insertion cassette using a polymerase chain reaction (PCR) protocol (described in detail in section 2.4.2). The samples analyzed with both criteria were taken from the first and last harvest of two SSC cultivations operated over 16 d, one with 25 h RT in the SVB and a second with 40 h RT in the SVB.

### Continuous production of MVA virus in a two-stage stirred tank bioreactor system

A bioreactor system consisting of two 1 L stirred tank bioreactors (Biostat B Plus, Sartorius) was established (similar to Frensing et al. 2013, Fig 1B). The first bioreactor (Cell Bioreactor, CB) was inoculated with AGE1.CR.pIX cells at 1×10^6^ cells/mL and operated at 37°C, 120 rpm with Rushton impellers, 40% oxygen saturation and 850 mL working volume (wv). Oxygen saturation was controlled using pulsed aeration with pure oxygen. The pH value was not controlled and only monitored during the batch phase to avoid values below 6.9. When the cell concentration in the CB reached at least 5.0×10^6^ cells/mL, 350 mL from the CB were transferred to the second vessel VB. Working volumes were corrected to 850 mL in CB and 440 mL in VB by adding fresh CD-U3 medium and continuous culture was initiated 2 h later and maintained without infection for 8 d. The dilution rate of CB (D_CB_) was as reported previously [6], and the dilution rate of VB (D_VB_) was 0.04 h^-1^ (25 h RT). Temperature of VB was 37°C, oxygen concentration was at 40–50% saturation, and the 440 mL wv was maintained with a dip tube. Before infection, a 1:1 dilution of VB was carried out, and the cultivation continued at 440 mL wv. MVA-CR19 virus was added to VB at an moi of 0.05. The peristaltic pumps used were Ismatec Reglo-Digital MS2/8-160 (Pump 1 and 2, Fig 1B; Cole-Parmer GmbH, Germany), and Watson Marlow 101U/R (Pump 3, Fig 1B; Waston-Marlow Fluid Technology Group, UK). Samples of 5–6 mL were taken twice a day from both vessels for measuring cell concentration, viability, off-line pH, metabolite concentrations, and virus concentration.

### Analytics

#### Cell concentration, viability, and metabolite concentrations

A ViCell^TM^ XR cell viability analyzer (Beckman Coulter GmbH, Germany) was used for determining AGE1.CR.pIX cell concentration and viability, with a standard deviation of 5% [28]. Glucose and lactate were determined to avoid media limitations using a BioProfile 100 Plus (Nova) analyzer; the relative standard deviation of the method is 1.9% for glucose and 10.5% for lactate, respectively.

#### Virus quantification assay, and polymerase chain reaction (PCR) protocol used for MVA-CR19.GFP virus stability analysis

The concentration of infectious virus particles was quantified by a TCID_50_ assay as described previously [29]. In addition, the GFP-derived TCID_50_ titer was obtained by measuring cell fluorescence using a fluorescence microscope (λ 495 nm, Axio Observer A1, Zeiss, Germany). The GFP-assay was carried out immediately before performing the regular TCID_50_ staining procedure. The PCR was carried out by mixing the infected cell suspension (80 μL) with 20 μL of QuickExtract DNA Extraction Solution 1.0 (Epicentre, USA) and heated to 65°C for 10 min and to 98°C for 5 min. Of this preparation, 4 μL were used in a PCR reaction in a final volume of 20 μL with 0.15 μL Taq polymerase (Qiagen, Germany), 200 nM of each primer, and 125 μM of each nucleotide. The sequence of the primer pairs that span deletion sites II, III and IV of the viral genome have been published previously [30]. The expected sizes of the amplification products are 354, 447, and 502 bp for wildtype virus deletion sites II to IV, and 1285 for deletion site III in MVA-CR19.GFP. Thermocycling was initiated with 94°C for 80 s, followed by 35 cycles of 94°C for 20 s, 55°C for 20 s and 72°C for 90 s, and terminated with 72°C for 5 min. Amplicons were separated by electrophoreses in 1.5% agarose gels.

### Productivity indicators of the cultivation systems

The productivity of batch, semi-continuous and continuous cultivation systems was determined based on two parameters: time yield (TY) and space-time yield (STY). The following equations were used for all three cultivation modes:

$${TY}_{t_{n}}=\frac{\sum_{t_{0}}^{t_{n}}\left({TCID50}_{H,t_{n}}\times V_{H,t_{n}}\right)}{t_{n}}$$ (6)

$${STY}_{t_{n}}=\frac{\sum_{t_{0}}^{t_{n}}\left({TCID50}_{H,t_{n}}\times V_{H,t_{n}}\right)}{\sum_{t_{0}}^{t_{n}}(V_{H,t_{n}})\times t_{n}}$$ (7)

Where t_n_ is the total operational time (initial batch phase plus virus production phase for semi-continuous and continuous), ${{TCID}_{50}}_{H,t_{n}}\;$ is the TCID_50_ of a harvest at time t_n_ (if no harvest is taken at time t_n_ its value is zero), and $V_{H,t_{n}}\;$ is the harvest volume collected at time t_n_. Eqs 6 and 7 were also used for estimating the productivity of a hypothetical batch process consisting of two parallel 645 mL bioreactors (8 d batch cycle). Considering possible mass transfer limitations for larger scales, the (maximum) productivity of a hypothetical batch cultivation was estimated using virus titers obtained in small-scale cultivations, i.e. the titers of BM-A, BM-B, and BM-C shown in Table 1. This hypothetical system was chosen as a comparison to the TSB system, because it represents the alternative given up when the decision of operating both vessels in continuous mode is taken (opportunity cost). From here onward this hypothetical process is referred as the batch process.

**Table 1:** Overview of the process parameters, virus titers and yields obtained in batch, semi-continuous, and continuous experiments.

Experiment ^a^	Cell Passage Number	Cell Conc. at toi [ˣ10^6^ cells/mL] ^i^	Medium Manufacturer	Virus	Dilution rates^b^	Stream F_3_ ^b^	RT in SVB or VB [h] ^c^	Volume SVB or VB [mL]	Day of Operation [d] ^d^	Maximum Virus Titer [virions/mL]	Total Number of Virions Produced [virions] ^e^	Total Harvest Volume [mL]	Time Yield [virions/h]	Space-time Yield [virions/(L h)]
BM-A	82	3.2	Biochrom	MVA-CR19	B	B	72	50	8.0	3E+08	2E+10	50	8.2E+07	1.6E+09
BM-B	41	2.5	Merck/Biochrom	MVA-CR19	B	B	72	50	8.0	1E+08	5E+09	50	2.6E+07	5.2E+08
BM-C	41	2.7	Merck/Biochrom	MVA-CR19	B	B	72	50	8.0	3E+07	2E+09	50	8.2E+06	1.6E+08
BM-average ^f^	-	-	Merck/Biochrom	MVA-CR19	B	B	72	50	8.0	1E+08	7E+09	50	3.6E+07	7.3E+08
2 Parallel batches^g^	-	-	Merck/Biochrom	MVA-CR19	B	B	72	1290	17.0	1E+08	4E+11	2580	8.9E+08	3.4E+08
2 Parallel batches ^h^	-	-	Merck/Biochrom	MVA-CR19	B	B	72	1290	26.0	1E+08	5E+11	3870	8.7E+08	2.2E+08
SM25-A	50	10.5	Biochrom	MVA-CR19	3·D_1_ = D_2_	F_3_ = F_3_	25	65	22.0	2E+09	2E+11	1136	3.7E+08	3.3E+08
SM25-B	90	12.9	Biochrom	MVA-CR19	3·D_1_ = D_2_	F_3_ = F_3_	25	65	22.0	2E+09	5E+11	1004	1.0E+09	1.0E+09
SM25-MOCK	82	-	Biochrom	MOCK	3·D_1_ = D_2_	F_3_ = F_3_	25	65	18.0	MOCK	MOCK	726	MOCK	MOCK
SM35-A	73	12.1	Biochrom	MVA-CR19	2·D_1_ = D_2_	F_3_ = F_3_	35	98	12.0	3E+08	2E+10	816	7.6E+07	9.3E+07
SM35-B	40	7.47	Merck/Biochrom	MVA-CR19	2·D_1_ = D_2_	F_3_ = F_3_	35	98	19.0	1E+09	2E+11	1157	4.1E+08	3.6E+08
SM35-C	40	4.42	Merck/Biochrom	MVA-CR19	2·D_1_ = D_2_	F_3_ = 1	35	65	18.0	3E+05	3E+07	649	7.4E+04	1.1E+05
SM64	73	11.8	Biochrom	MVA-CR19	1·D_1_ = D_2_	F_3_ = F_4_	64	198	12.0	6E+08	6E+10	1084	2.0E+08	1.8E+08
SG25	69	5.72	Merck/Biochrom	MVA-CR19.GFP	3·D_1_ = D_2_	F_3_ = F_4_	25	62	19.5	1E+08	3E+10	1148	6.0E+07	5.2E+07
SG40	69	6.01	Merck/Biochrom	MVA-CR19.GFP	2·D_1_ = D_2_	F_3_ = F_4_	40	120	19.5	6E+09	5E+11	1208	1.0E+09	8.7E+08
T25	50	9.19	PAA	MVA-CR19	3 D_1_ = D_2_	F_3_ = F_3_	25	440	21.7	6E+08	6E+11	7100	1.2E+09	1.7E+08

^a^ T = Two-stage continuous bioreactor; S = semi-continuous small scale cultivation; B = Batch; M = MVA-CR19 strain; G = MVA-CR19.GFP strain; XX = XX hours (25 h,35 h or 64 h) of residence time in the VB or the SVB.

^b^ F_3_ = D_1_ (V_2_ + V_1_)—F_1_ with D_1_ the dilution rate of CB or SCB, V, thevolume of each vessel, and F, the flow rate.

^c^ RT = residence time; VB = Virus Bioreactor; the value shown for batch cultures corresponds to the harvest time (h p.i).

^d^ considering a batch with 4 days of cell growth in all processes, 3 days of virus production and 1 day for cleaning and sterilization.

^e^ this value corresponds to the total number of virions produced after adding the virus collected from each harvests. This was calculated by multiplying the TCID_50_ of each harvest by its volume.

^f^ the average TCID_50_ titer of batch A, B and C was estimated to be 1x10^8^ virions/mL

^g ^two parallel 645 mL batch bioreactors; calculations were carried out assuming 2 batch-cycles, because it approaches the operational time of the SSC cultivations (2 weeks). Note: the TY is valid only for a specific cultivation scale, while the STY is independent of the cultivation scale. The complete time course of such a process is shown in Fig 6.

^h^ two parallel 645 mL batch bioreactors; calculations were carried out assuming 3 cycles (26 d), because it approaches the operational time of the TSB experiment (T25; 3 weeks). The complete time course of such a process is shown in Fig 6.

^i ^cell concentration at time of infection.

## Results and discussion

### Batch cultivations

#### MVA virus titers and productivity in batch

Cells were seeded in shaker flasks at a concentration of 0.8×10^6^ cells/mL and grew in batch mode for 3–4 d until 5.0×10^6^ cells/mL were reached. For the MVA-CR19 virus strain used for infection, TCID_50_ titers of 3.0×10^8^, 1.0×10^8^ and 0.3×10^8^ virions/mL were obtained at 72 h post infection, as summarized in Table 1 (experiments BM-A, B and C). Based on the average TCID_50_ titer of 1.4×10^8^ virions/mL at time of harvest (toh; defined as the time with the highest virus titer), the productivity of the hypothetical batch process (see productivity indicators section) was determined as 8.9×10^8^ virions/h (TY^B^) and 3.4×10^8^ virions/(L h) (STY^B^) for 17 d of operation (2 batch cycles). For 26 d of operation (3 batch cycles) this corresponds to 8.7×10^8^ virions/h and 2.2×10^8^ virions/(L h) for TY and STY, respectively (Table 1).

**Fig 2:**
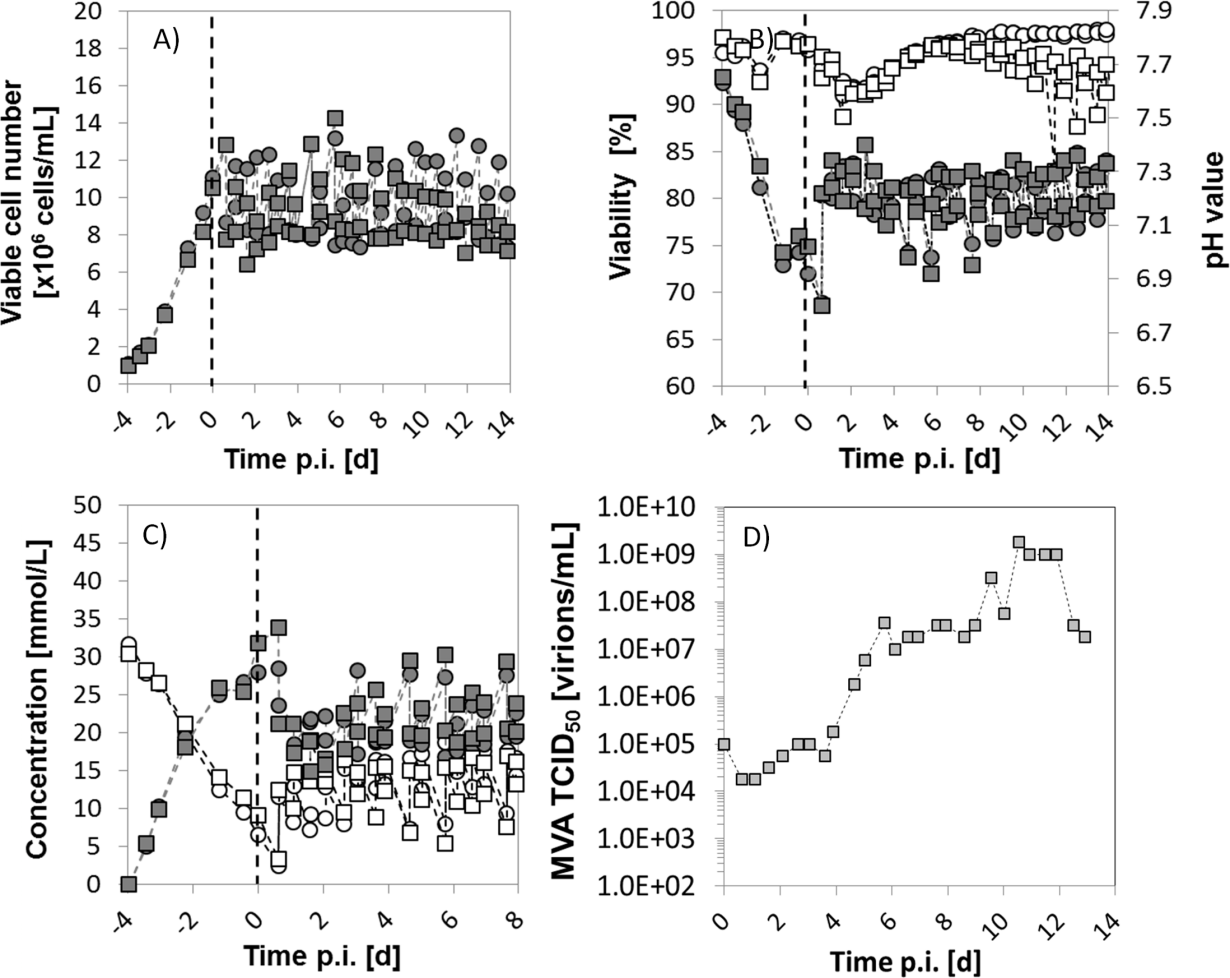
Semi-continuous propagation of MVA-CR19 virus in a two-stage system using shaker flasks (only one representative cultivation shown; experiment SM25-A, Table 1. A) Concentration of viable AGE1.CR.pIX cells in SCB (circles) and SVB (squares). B) Viability (white) and pH value (grey) of SCB (circles) and SVB (squares). C) Concentration of glucose (white) and lactate (grey) in SCB (circles) and SVB (squares). D) MVA TICD_50_ titers of SM25-A; the dashed line represents the time of infection. The first harvest was carried out 12 h post infection. SCB and SVB, small-cell and virus bioreactors.

MVA-CR19 virus titers obtained in these 50 mL batch experiments were similar to those previously described for MVA wild type virus replicated in AGE1.CR and AGE1.CR.pIX cells [31]. In particular, these experiments confirmed that the new genotype MVA-CR19 efficiently replicates in single cells and does not require cell agglomeration at the time of infection [24]. Taking into account the initial batch phase of the continuous experiment T25 (Fig 2A), where cell concentrations up to 5×10^6^ cells/mL were achieved in a 1 L bioreactor in batch mode, it should also be possible to obtain high virus titers at larger batch volumes.

### Semi-continuous cultivation

#### Cells and MVA virus propagation in the semi-continuous system

To start the cultivation, AGE1.CR.pIX cells were seeded at a concentration of 0.8×10^6^ cells/mL in SCB and SVB and maintained in batch mode for 3–4 d. Results of one representative cultivation are shown in Fig 2A, 2B, 2C and 2D (experiment SM25-A, Table 1). After the initial batch phase, medium replacements at regular intervals were initiated (12–15 h) and a semi-continuous steady-state was successfully obtained for at least 2 weeks. Viable cell concentrations in the SCB fluctuated in a range of 8–12×10^6^ cells/mL with viabilities above 90% (Fig 2B). No limitation of glucose was observed, and levels of lactate were about 20 mmol/L, as shown in Fig 2C.

SVB was infected with MVA virus after 3–4 d of batch growth, and the semi-continuous mode with harvesting was started 12 h p.i. The cell concentration initially also fluctuated in a similar range as for the SCB, but then fluctuations decreased as a consequence of progressing virus replication. Cell viabilities of 90% or more were observed in SVB for the first days, but dropped after 12 d (Fig 2B). The virus titers increased for about 4–5 d before a stationary phase was achieved (Fig 2D). Final TCID_50_ values between 1×10^7^−1×10^9^ virions/mL, were obtained that are similar to those of batch cultivations and published data [24, 31]. In contrast to influenza A virus cultivations in continuous cultures, which oscillated by 6 orders of magnitude [6], only a low level of random fluctuations in MVA-CR19 titers were observed that did not exceed two orders of magnitude. For example, SM25-A (grey squares, Fig 2D) oscillated between 1×10^7^−1×10^9^ virions/mL. Taking into account the results obtained for MVA-CR19.GFP stability (see following sections), these oscillations are most likely due to variations in the cell concentration in the SVB and measurement errors of virus titrations.

#### Impact of process parameters on MVA virus yields: Residence time and addition of fresh medium in the virus bioreactor

**Fig 3:**
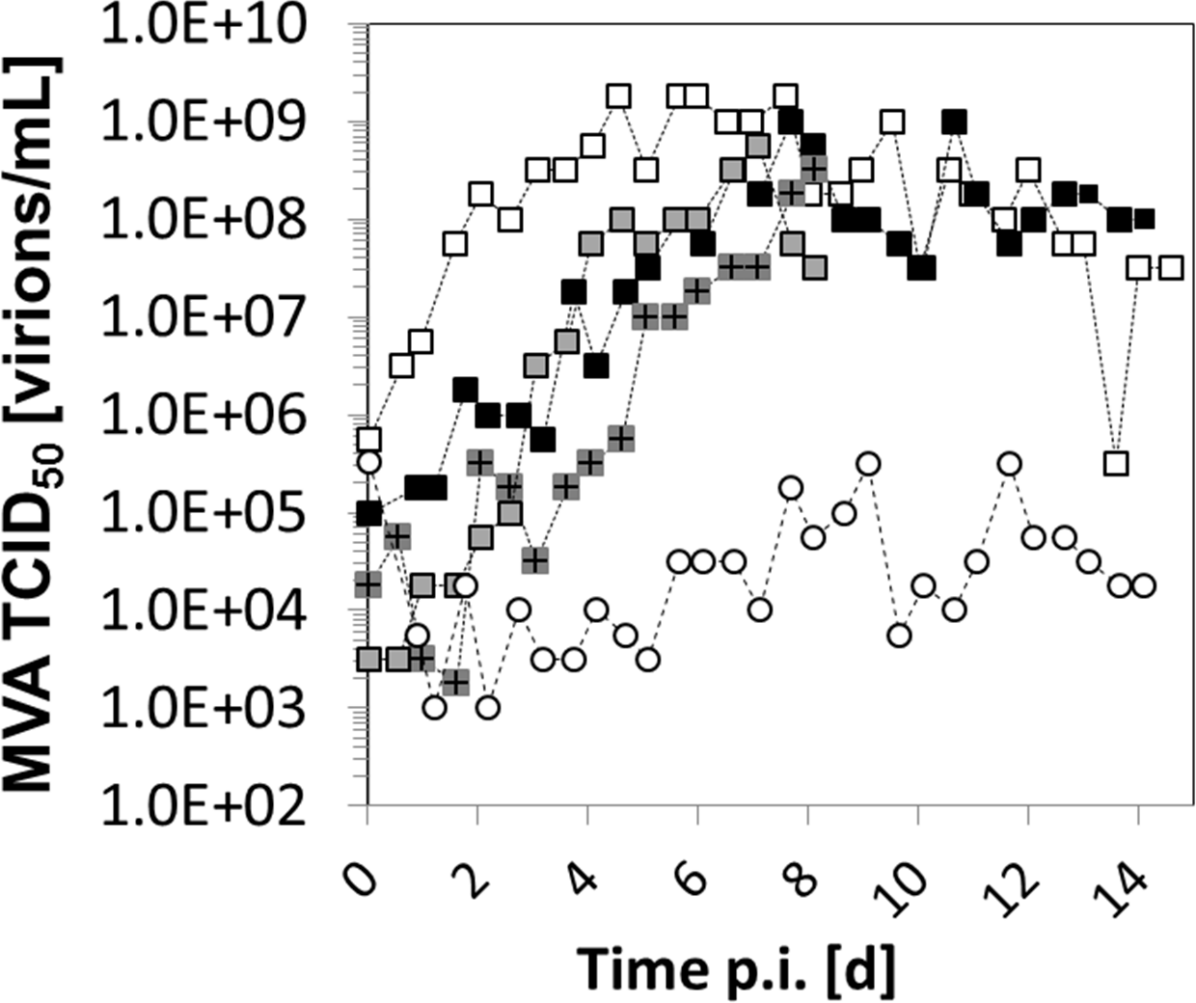
Semi-continuous propagation of MVA-CR19 virus at three different residence times (25, 35 and 64 h) in the virus vessel (SVB). MVA TCID_50_ titers of the semi-continuous experiments (squares) SM25-B (25 h, white), SM35-A 35 h, grey with +), SM35-B (35 h, black), and SM64 (64 h, grey). One semi-continuous experiment, SM35-C (white-circles), was carried out without medium replacement in the SVB.

To evaluate if the virus yields can be increased by changing the residence time, SSC experiments with 25, 35 and 64 h RT in the SVB were carried out (Fig 3). The moi of 0.05, used in all experiments, led to initial virus concentrations of 0.1–1.0×10^5^ virions/mL. The TCID_50_ titers showed the typical pattern of virus replication, an initial increase followed by a stationary phase. Depending on the RT in the SVB, the increase in virus titers differed significantly. With the lowest RT (SM25-B, 25 h), the increase was fast and high titers were achieved within 2 d (≥1×10^8^ virions/mL, Fig 3). Surprisingly, virus increase was delayed with higher RT (SM35-B and SM64, 35 and 64 h). Nevertheless, for both cultivations final virus titers in the order of 1×10^8^ virions/mL were obtained.

Also, to test whether high virus yields can be obtained by reducing the amount of fresh medium added to SVB, one experiment was performed without the addition of fresh medium into the virus vessel (SM35-C; Table 1, Fig 3). With fluctuations in TCID_50_ titers not exceeding 1×10^5^ virions/mL, this cultivation resulted in the lowest virus titers among all experiments. This is in agreement with previous studies (performed with influenza A virus, however) which showed that addition of fresh medium at time of infection is usually required for obtaining high virus yields in batch processes [32]. Despite the differences during the initial increase in virus titers (8–10 d), all SSC cultivations achieved comparable maximum TCID_50_ titers in the stationary phase with fluctuations of less than 2 log units. Taking additionally into account the results of the SSC cultivations, a RT of 25 h was therefore used to scale up the virus bioreactor in the continuous cultivation.

#### Productivity of the semi-continuous experiments

As a general tendency, a higher productivity was obtained for experiments with lower RT in SVB. The TY was 1.0×10^9^, 4.1×10^8^, and 2.0×10^8^ virions/h for the experiments SM25-B, SM-35-B and SM64, respectively (Table 1). The best STY was 1.0×10^9^, 3.6×10^8^, and 1.8×10^8^ virions/(L h) for experiments SM25-B, SM-35-B and SM64, respectively. These values were in the same order of magnitude as the batch process operated 8 d (5.2×10^8^ virions/(L h), 1 cycle) and 16 d (2.5×10^8^ virions/(L h), 2 batch cycles), respectively. Accordingly, the productivity for SSC systems is similar or better than for batch processes. It has to be taken into account, however, that a batch process is more efficient than SSC for low volume MVA vaccine production (single batch campaigns).

#### Analysis of MVA-CR19.GFP virus stability in semi-continuous cultivations at 25 h and 40 h of residence time

**Fig 4:**
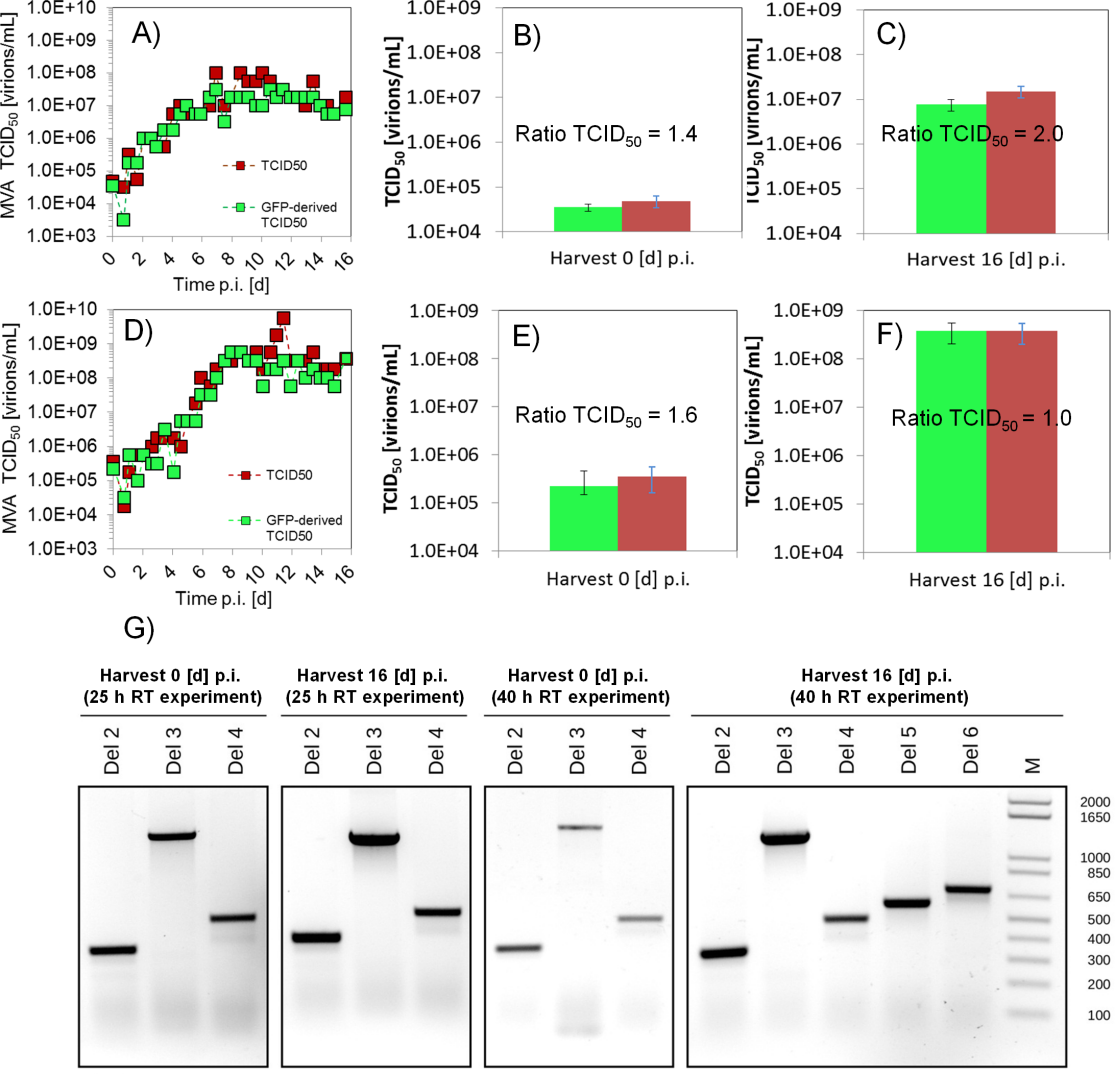
Genetic stability analysis of MVA-CR19.GFP virus for 16 d of semi-continuous cultivation. Two different RT in SVB were analyzed. Fig A, B and C correspond to experiment SG25 (25 h RT in SVB) and Fig D, E, and F to experiment SG40 (40 h RT in SVB). A) TCID_50_ (red) and GFP-derived TCID_50_ (green). B) TCID_50_ (red) and GFP-derived TCID_50_ (green) at 0 d p.i. C) TCID_50_ (red) and GFP-derived TCID_50_ (green) at 16 d p.i. D) TCID_50_ (red) and GFP-derived TCID_50_ (green). E) CID_50_ (red) and GFP-derived TCID_50_ (green) at 0 d p.i. F) TCID_50_ (red) and GFP-derived TCID_50_ (green) at 16 d p.i. Error bars: mean and standard deviation of three technical replicates. G) PCR analysis of the deletion segments 2, 3, 4, 5 and 6 (Del 2–6) of MVA-CR19.GFP virus, and DNA ladder in the range of 100–2000 bp (M). The first and last harvest of experiments SG25 (two boxes on the left side) and SG40 (two boxes on the right side) were analyzed.

In order to investigate the genetic stability of the MVA virus over extended cultivation periods (i.e. the risk to lose the recombinant GFP protein), the MVA-CR19.GFP virus was propagated for 16 d in SSC, and the TCID_50_ as well as the GFP-derived TCID_50_ were determined (25 h RT, Fig 4A; 40 h RT, Fig 4D). For both RT experiments, the time course and the absolute values of both TCID_50_ titers were similar over the whole cultivation period. At 25 h RT, a Ratio-_TCID50_ of 1.4 and 2.0 was obtained for the first and the last harvest, respectively (Fig 4B and 4C). At 40 h RT, the ratios were 1.6 and 1.0, respectively (Fig 4E and 4F). Considering that the error range of the TCID_50_ assay is a least 0.3 log units due to the dilution steps chosen for titrations, this variation is not significant. The Ratio-_TCID50_ of 1.0 and 2.0 after 16 d of culture in both RT experiments suggests that the MVA virus is genetically stable. Interestingly, the MVA-CR19.GFP virus titers were higher for a RT of 40 h rather than 25 h. One potential explanation may be that the incorporation of a recombinant insert may have slightly prolonged the virus replication cycle so that 25 h RT may be too short for efficient propagation in the SVB.

In addition, the genetic stability of MVA-CR19.GFP virus was analyzed using PCR of the first and last harvest of both SSC cultivations. For the two RT experiments, the GFP insertion cassette amplified by this method was visible in the deletion segment 3 (Del 3) of the first (0 d p.i.) and last harvest (16 d p.i.) with a size of 1428 kbp (Fig 4G). The fact that this band was visible in the first and last harvests, and that no smaller gene fragments where detected in this cultivation, supports the finding regarding the genetic stability of MVA-CR19.GFP discussed above. For longer cultivations and for the production of other recombinant antigens, these results have to be confirmed. In general, however, MVA-CR19 seems to be an excellent candidate for continuous viral vector production.

### Continuous cultivations

#### Cell and MVA virus propagation in the continuous bioreactor

**Fig 5:**
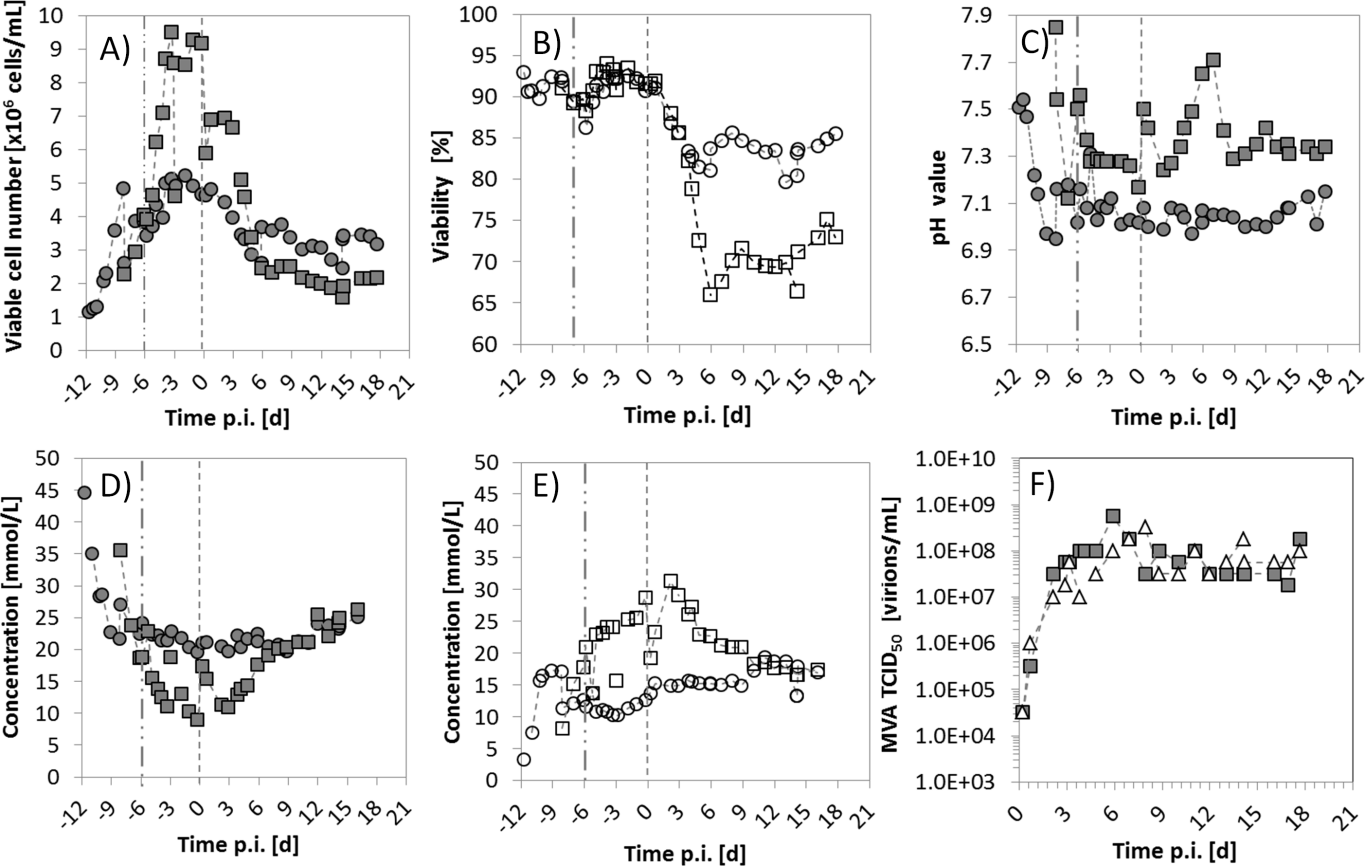
Continuous cultivation of MVA-CR19 virus in a two-stage stirred tank bioreactor (TSB) system. Data of CB (circles) and VB (squares) are shown. A) Viable cell concentration. B) Cell viability. C) pH value. D) Concentration of glucose. E) Concentration of lactate. F) TCID_50_ titers of MVA-CR19 virus in VB (grey squares) and in the harvest (white triangles). The dotted-dashed vertical line at -7 h p.i. represents the start of the continuous culture in both vessels. The dashed line at 0 d p.i. represents the time of infection of VB.CB and VB, cell and virus bioreactors.

The continuous bioreactor system (experiment T25, Table 1) was successfully operated for 30 d, as shown in Fig 5. During the startup of the process (-12 d to -8 d), the cell concentration in the CB reached values of 5.0×10^6^ cells/mL in batch operation with cell viabilities well above 90% (Fig 5A and 5B), and μ_max_ of 0.0150 h^-1^. Both, the maximum cell concentrations and the μ_max_ are in accordance with results reported previously by Lohr et al (2009). Immediately before the continuous culture was initiated, a concentration of about 2.5×10^6^ cells/mL was reached in both vessels by 1:1 dilution with fresh medium. Once the continuous operation was started, the cell concentration in the CB increased initially to 5.0×10^6^ cells/mL, and finally decreased to 3.0×10^6^ cells/mL with 85% viability. Nevertheless, cells could be maintained stable at these viabilities for the rest of the experiment. Also, the continuous process was maintained without infection of VB for the next 8 d (-7 d to 0 d p.i., Fig 5A), in order to evaluate cell growth in the second vessel. Finally, the cells concentration in the VB reached up to 9.0×10^6^ cells/mL, well above the anticipated 5.0×10^6^ cells/mL of CB. One possible explanation is that the dip tube of the VB, used to extract the harvest, might have acted as a settler as it had a relatively low withdrawal rate and a high internal diameter as reported previously [33]. Achieving high cell concentrations, the glucose concentration in the VB dropped to about 10 mmol/L (Fig 5C). Therefore the 50% of medium of the VB was exchanged before infection with MVA-CR19 virus. After the infection (moi 0.05), the cell concentration and the viability in VB decreased to 5.0×10^6^ cells/mL and 70%, respectively, as expected from the SSC experiments described above. After three days of continuous cultivation, the TCID_50_ reached values close to 1.0×10^8^ virions/mL, and a stable MVA production was maintained for 18 d p.i. with a maximum titer of 6.0×10^8^ virions/mL, very similar to batch cultivations [31]. A total number of 6.0×10^11^ virions were collected from the harvest, with a total production volume of 7.1 L, which corresponds to an average TCID_50_ of 9×10^7^ virions/mL (also see data for T25 in Table 1). As before, the virus titers fluctuated in a range not larger than 1 log unit (see SSC experiments). This low level of fluctuations of infectious titers over process time together with the observed genomic stability of MVA-CR19.GFP suggests that interference by defective particles [34, 35] is not a phenomenon that limits continuous MVA replication.

In Fig 5D, the time course of the TCID_50_ of the VB and the harvest bottle are depicted. Note that, while the TCID_50_ of VB represents the virus concentration in the vessel at a given time point, the TICD_50_ of the harvest represents the value that results from accumulating virus production over a period of 8–12 h. During this time period, part of the MVA virus could be inactivated by host cell hydrolases released to the supernatant. Thus, the slightly lower TCID_50_ titers obtained in the harvest compared to the VB might be closer to the real TCID_50_ that will be obtained in a continuous process with a harvest bottle connected to the VB.

Interestingly, the TSB results resembles some of the results obtained with the semi-continuous cultivation system. Experiments SM25-A and SM25-B were performed with a RT in SVB identical to the VB of the TSB system. Virus titers from SM25-A (Fig 2D) showed a similar pattern as the values of the TSB system (Fig 5F) from day 4 p.i. onwards, reaching similar final titers. The second experiment, SM25-B (white squares, Fig 3), was even closer to the virus dynamics obtained with the TSB system because the harvest volume was correctly calculated using Eq 4 (a wrong calculation of the first harvest volume of SM25-A diluted the virus below 1×10^5^ virus/mL between 0–4 d p.i.). This confirms that SSC could serve as a scale-down model of the TSB system, and is in line with previous work performed by Van Lier et al. who have demonstrated for baculovirus *A*. *californica* that semi-continuous cultivations can approximate continuous two-stage cultures [27]. In addition, it confirms studies regarding the use of simple non-instrumented batch and semi-continuous cultures for the design and optimization of continuous animal cell cultures [26]. Overall, our results show that these concepts can even be applied for two-stage virus cultivations systems using shaker flasks.

#### Productivity of the continuous process

**Fig 6:**
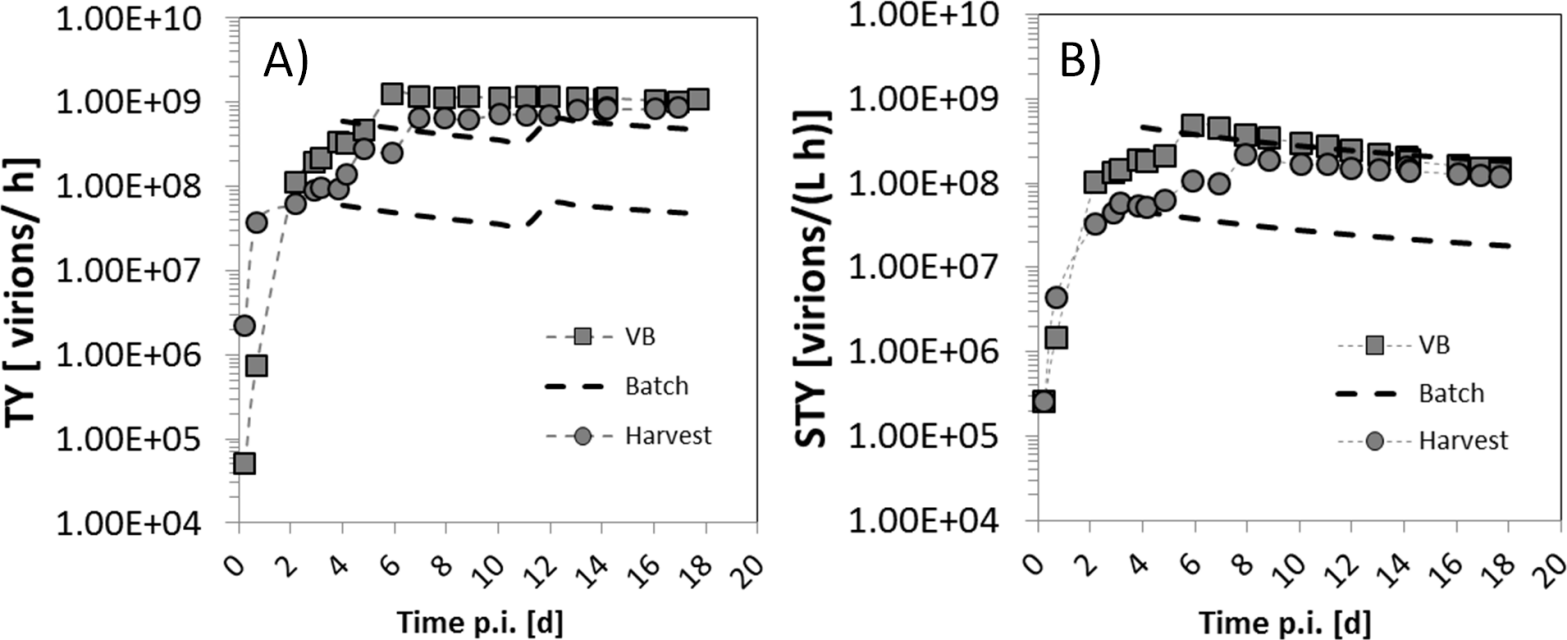
Productivity of the two-stage stirred tank bioreactor (TSB) system (1290 mL wv; 0.29 mL/min) compared with a hypothetical batch process (645 mL each vessel, 1290 mL wv). A) TY of the continuous cultivation based on TCID_50_ values by sampling the VB (squares) and the harvest vessel (circles), versus TY of the batch process (dashed lines; upper and lower lines estimated, assuming a maximum TCID_50_ at time of harvest of 1×10^8^ and 1×10^7^ virions/mL, respectively). B) STY of the continuous cultivation compared to the batch process (same symbols as in Fig A). It is further assumed that the cell growth phase of the batch and the continuous cultivation were identical, and that both vessels of the batch process were harvested at day 3 and day 12 post infection. TY, time yield; STY, space-time yield; VB, virus bioreactor.

The TY of the TSB system was 1.2×10^9^ virions/h, and the STY was 1.7×10^8^ virions/(L h) for a total of 21.7 d of operation (17.7 d of virus propagation). The time course of TY and STY over the production time is shown in Fig 6A and 6B. As a comparison, data of an hypothetical batch process (10^7^ virions/mL and 10^8^ virions/mL at toh) are also included. This figure shows that both the TY and the STY of the TSB started below the productivity of a batch. After 7 days, the productivity of the TSB approached the batch process and remained constant. In other words, the TSB system can be equally or more efficient than a batch production system, if more than two batch-cycles are performed (see detail in Table 1).

## Conclusions

Continuous and semi-continuous production of MVA virus was evaluated and compared against batch cultivations using MVA-CR19 and MVA-CR19.GFP virus isolates that allow efficient replication in non-agglomerated avian AGE1.CR.pIX suspension cells. A small-scale approach, using a two-stage semi-continuous cultivation (SSC) system, was used to support the establishment of a two-stage continuous stirred tank bioreactor (TSB) system that showed stable production of cells over 2–3 weeks of cultivation.

A higher residence time in the SSC system resulted in a higher delay before virus titers increased, but had little impact on the final titers of the MVA-CR19 isolate in the stationary phase. A critical parameter for high yields appears to be the incorporation of a stream of fresh medium in the virus bioreactor, to remove spent medium including compounds inhibiting virus replication and to avoid limitation of cell-relevant metabolites during virus replication.

Evaluation of continuous production of MVA was facilitated with the SSC system because high virus titers were obtained throughout the process interval without oscillations of infectious units at different residence times. The stable titers suggest that a “von Magnus effect” due to formation of DIPs is highly unlikely for MVA. This observation is also consistent with the results from assays for maintenance of recombinant inserts by titration against the fluorescence GFP transgene and by PCR analysis. These assays demonstrated that MVA is stable for at least 16 d of continuous production.

A scale up to a TSB system for MVA-CR19 virus propagation was successful and showed high TCID_50_ titers, again with only small random fluctuations over 18 d of virus production and with productivity indicators comparable to repeated-batch cultivations. However, titers appeared to be lower for the recombinant virus that expresses GFP if the RT was only 25 h. This observation may indicate that MVA-CR19.GFP may have longer replication cycles under the chosen conditions and that the RT has to be adjusted accordingly for scale-up.

Overall, our results demonstrate that at least some recombinant MVA virus strains can be stably and efficiently propagated in TSB systems designed for continuous and semi-continuous production of MVA-based recombinant vaccines and viral vectors.

## Abbreviation

CB: cell bioreactor
DIPs: defective interfering particles
moi: multiplicity of infection (ratio of the number of infectious virions to the number of cells), [–]
MVA: modified vaccinia Ankara
RT: residence time, [h]
SCB: semi-continuous cell bioreactor
SSC: small-scale semi-continuous cultivation
STR: stirred tank bioreactor
SVB: semi-continuous virus bioreactor
STY: space time yield, [virions/(L h)]
toi: time of harvest, [h]
TSB: two-stage stirred tank bioreactor
TY: time yield, [virions/h]
VB: virus bioreactor
